# Undeveloped green space and free-time physical activity in 11 to 13-year-old children

**DOI:** 10.1186/s12966-015-0187-3

**Published:** 2015-02-21

**Authors:** Ian Janssen, Andrei Rosu

**Affiliations:** School of Kinesiology and Health Studies, Queen’s University, Kingston, ON Canada K7L 3N6; Department of Public Health Sciences, Queen’s University, Kingston, ON Canada

**Keywords:** Child, Motor activity, Environment, Nature, Trees, Health surveys

## Abstract

**Background:**

Research on the association between the physical environment and physical activity in children has focused on built and developed features or total green space. The impact of natural, undeveloped green spaces is unknown. The objective of this study was to determine whether the presence of undeveloped green spaces in the home neighborhood are associated with physical activity in 11 to 13-year-olds.

**Methods:**

This was a cross-sectional study of grade 6 to 8 urban residing Canadian students who participated in the 2009/10 Health Behaviour in School-Aged Children survey. Children self-reported the frequency they participated in physical activity in their free-time outside of school hours. Geographic Information Systems (GIS) were used to assess the proportion of land area within 1 km of participants’ homes that was devoted to publicly accessible meadows (i.e., field vegetated primarily by grass and other non-woody plants) and treed areas (i.e., field vegetated primarily by trees and shrubs). Ordinal logistic regression models were used to examine the relationships between the undeveloped green space areas and free-time physical activity. Several intrapersonal, family, and neighborhood environment factors were controlled for in these regression models.

**Results:**

The proportion of neighborhood land covered by meadows was not associated with the physical activity outcome (p > 0.6). However, the proportion of neighborhood land covered by treed areas was independently associated with the physical activity outcome (p = 0.02). For each additional 5% increase in the proportion of neighborhood land covered by treed areas there was a corresponding 5% increase (95% confidence interval: 1-10% increase) in the relative odds of increasing free-time physical activity outside of school hours.

**Conclusions:**

The physical activity levels of 11 to 13-year-old children was associated with the amount of space in their home neighborhood devoted to treed areas.

## Background

Objectively measured physical activity data from Canada indicate that less than 1 in 10 children meet the public health guideline of 60 minutes per day of moderate-to-vigorous physical activity [1]. The physical inactivity crisis has not gone unnoticed by the research community. Indeed, an abundance of studies have attempted to unravel the determinants of a physically inactive lifestyle among children [2-5]. Of particular note is the proliferation of studies in the past decade examining the association between the physical environment and physical activity [3,4].

The relationship between the physical environment and physical activity is complex. “Walkability”, a physical environment feature that promotes physical activity amongst adults [6], does not do so in children. In fact, several studies have reported that walkability in the home neighborhood is negatively associated with children’s physical activity [7-9]. Conversely, physical environment features that reflect poor walkability, such as a high density of cul-de-sacs, are positively associated with children’s physical activity [7-9]. Cul-de-sacs could be an important outdoor play area for children, and the “playability” of the home neighborhood environment may be more relevant for young people that its walkability [8-10].

The playability of a neighborhood could reflect physical environment features that are built and developed, such as cul-de-sacs, city parks and playgrounds, and yards at home. It could also reflect physical environment features that are natural and undeveloped, such as meadows and treed areas. Research on the association between the physical environment and physical activity has focused on built and developed features [3,4]. While evidence suggests that total green space (including developed and undeveloped green space areas) may promote physical activity [11], the specific impact of natural, undeveloped green spaces is unknown. Therefore, the objective of this study was to determine whether the presence of undeveloped green spaces in the home neighborhood are associated with physical activity in children. This study focused on 11 to 13-year-old children, as this is an age when children should be old enough to play outdoors unsupervised, but (for some) not yet at the age where outdoor active play is not a relevant form of physical activity.

## Methods

### Data sources and study sample

This study was based on the cross-sectional 2009/10 Canadian Health Behaviour in School-Aged Children Survey (HBSC), which consists of two components: (1) a classroom-based health survey conducted on a representative sample of children in grades 6 to 10, and (2) GIS measures of the environment in the neighborhoods surrounding the HBSC participants homes.

The 2009/10 HBSC is a cross-national survey conducted in affiliation with the World Health Organization. This study was limited to the Canadian sample. The survey covered several aspects of health, health behaviors, and physical and social determinants of health. The Canadian sample was designed according to the international HBSC protocol [12]. The sampling strategy followed a systematic multi-stage cluster technique, whereby individual students are nested in school classes that are nested within schools and school boards. The 2009/10 Canadian HBSC included 26,078 students from 436 schools, with distributions reflecting the distribution of Canadians in grades 6 to 10 (approximate age range 11 to 15-years-old). All provinces and territories in Canada participated with the exception of Prince Edward Island and New Brunswick. Students enrolled in private, special needs, or home schools, as well as incarcerated youth, were excluded; combined they contribute to <10% of the 11 to 15-year-old population. Consent was obtained and provided by school boards, individual schools, participants, and their parents/guardians. Ethics approval was obtained from the General Research Ethics Board of Queen’s University and Health Canada’s ethics board.

Given the objective of this study, an a priori decision was made to examine children and not adolescents. Therefore, 6,667 grade 9 and 10 high school students were excluded from the original sample of 26,078 grade 6 to 10 students. Because postal codes were used as proxies of the home address when obtaining the neighborhood environment measures (explained below), of the 19,411 grade 6 to 8 children, 8,032 with missing or invalid postal code data were excluded. Next, we excluded 2,351 students from rural areas as postal codes cover a large geographic area in rural areas; in Canadian urban settings postal codes cover a small geographic area (i.e., 1 or 2 blocks) [13]. An additional 3,524 participants were excluded because Google Earth street view images were not available for their neighborhood, and these images were needed to measure the undeveloped green spaces. Finally, 366 participants were excluded because they did not report their physical activity or covariate data in the questionnaire. The final sample consisted of 5,138 grade 6 to 8 students who were approximately 11 to 13-years-old.

### Undeveloped green space in the home neighborhood (exposure)

A 1 km radius circular buffer around participants’ homes, as measured from the geographic center of their postal code, was used to define their home neighborhoods. A 1 km distance represents a 10 to 15 minute walking time [14]. Previous research supports this as an appropriate buffer size for children. The majority of children are not allowed to not travel >1 km from home unsupervised [15]. Measures of street connectivity obtained for a 1 km buffer were more consistently related to physical activity in youth than similar measures obtained at larger buffer sizes [16]. Furthermore, in the Canadian HBSC fast food restaurant measures obtained for a 1 km buffer are more strongly related to eating behaviors than measures obtained for smaller and larger buffer sizes [14].

The percentage of land area comprised of undeveloped and publicly accessible green space was determined for each neighborhood. The undeveloped green space consisted of meadows and treed areas. A meadow is defined here as a field vegetated primarily (>50%) by grass and other non-woody plants. A treed area is defined here as a field vegetated primarily (>50%) by trees and shrubs. Green areas that were not publicly accessible (e.g., fenced off, industrial property, yards at peoples’ homes), that were covered by water (e.g., lakes, wetlands), and that consisted of developed green space (e.g., parks with amenities such as a playground, sports fields, school grounds) were excluded. The exclusion of these areas essentially left meadows and treed areas as the remaining green spaces.

To process for measuring undeveloped green space started in ArcGIS version 10.2 software (Esri, Redlands, CA). First, we created 1 km circular radius buffers around the central point of each participant’s postal code to define their neighborhood. Next, for each of the participating schools we created a polygon that covered all of the neighborhood buffers for that school’s participating students. These polygon shapes were exported into Google Earth (Google, Mountain View, CA) where they were added as a layer on top of Google Earth satellite images. A layer of developed park spaces was also exported from ArcGIS into Google Earth to help distinguish between developed and undeveloped green space. Once these layers were superimposed on the satellite images in Google Earth, potential undeveloped meadows were identified by carefully inspecting the satellite images. These potential undeveloped meadows were then inspected at the ground level using the Google Earth street view tool. Areas that were confirmed as meadows were then outlined with a polygon shape on the satellite images using the Google Earth editing tools. This process was repeated until all of the meadows in the larger polygon were identified, and then repeated to identify the treed areas. Once completed, the newly developed layers that contained the smaller polygons for the meadows and treed areas were exported into ArcGIS. ArcGIS was then used to calculate the land coverage area of these undeveloped green spaces and the total land area (not including water and wetlands) separately for each participant’s neighborhood buffer. We then calculated the percentage of total land area that was made up of the undeveloped green spaces.

All of the undeveloped green space measures were obtained by a single rater. To determine the intra-rater reliability of the undeveloped green space measures, that rater completed the measures a second time, several weeks after obtaining the initial measures, for 1,404 participants from 33 schools. There was excellent agreement between the land coverage values for the first and second measures with correlation coefficients of r = 0.99 for meadows and treed areas.

### Physical activity in free-time outside of school (outcome)

Responses to the question “Outside school hours: how often do you usually exercise in your free time so much that you get out of breath or sweat?” were used to measure physical activity in free-time outside of school hours. Ordinal responses to this question were: “Never”, “Less than once a month”, “once a month”, “once a week”, “2 to 3 times a week”, “4 to 6 times a week”, and “Every day”. This question has been a mandatory item in the international HBSC survey dating back to the 1989/1990 cycle.

### Confounding variables

Variables considered as potential confounders as self-reported in the HBSC were gender, grade, race (white or other), number of parents in the household (single or dual), perceived neighborhood safety (assessed with the question “*It is safe for younger children to play outside during the day”* with 5 ordinal responses), and perceived family wealth (assessed with the question: *“How well off do you think your family is?”* with five ordinal responses). GIS measured confounders included average income in the neighborhood as obtained from the 2006 Census of Population, the number of recreational facilities in the neighborhood, the proportion of neighborhood land area made up of developed park and playground space, and the percentage of the total road distance within the buffer that was comprised of low speed roads (i.e., speed limit ≤50 km/h). These GIS measures were obtained in the 1 km neighborhood buffers and are described elsewhere [8]. Finally, we considered whether the survey was administered in the winter (December-March), fall (September-November), or spring (April-June).

### Statistical analysis

Analyses were performed in SAS version 9.3 (SAS Inc., Carry, NC) and accounted for the sample weights and clustered nature of the data. Distributions of key variables were characterized using conventional descriptive statistics. Spearman correlations were used to explore the relationships between the physical environment measures. Bivariate ordinal logistic regression models were initially used to describe the relationships between the undeveloped green space exposures and confounders with the physical activity outcome. This was followed by multivariate ordinal logistic regression models that included the primary exposure variables and all of the confounders that were related (p < 0.1) to the outcome in the bivariate models. With the exception of gender, race, parents in household, and winter season, all of the exposure and confounding variables were entered into the ordinal logistic regression models as continuous variables. The odds ratios and 95% confidence intervals from these models are expressed as follows: per 5% change in the proportion of land coverage for the undeveloped green space and developed park space variables, per each grade, per one unit change in ordinal responses for the perceived family wealth and perceived neighborhood safety variables, per $10,000 for average household income, per each additional neighborhood recreational facility, and per 10% change in the proportion of neighborhood road distance made up of low speed roads. Analyses indicated that there were no age or gender interactions; therefore, all participants were included in the same regression models.

## Results

A description of the participants is provided in Table 1. By design, there were comparable number of boys and girls and grade 6, 7, and 8 students. The majority of participants were White, perceived their family as being quite or very well off, lived with two parents, and agreed or strongly agreed that it was safe for children to play outside in their neighborhood. Only 23% of the participants indicated that they engaged in physical activity in their free-time outside of school hours on a daily basis.

**Table 1:** **Descriptive characteristics of participants as obtained in the Canadian health behaviour in school-aged children survey**

**Variable**	**N**	% of total*
**Gender**		
Boys	2376	47.6
Girls	2762	52.4
**Grade**		
6	1632	32.0
7	1718	33.1
8	1788	34.9
**Race**		
White	3610	71.9
Other	1528	28.1
**Perceived family wealth**		
Not at all well off	159	2.7
Not very well off	348	6.6
Average	1648	31.8
Quite well off	1644	32.2
Very well off	1344	26.8
**Parents in household**		
Single parent	1119	21.3
Dual parent	4019	78.7
**Perceive neighborhood as being safe for children to play outside**		
Strongly disagree	130	2.7
Disagree	272	5.8
Neither agree or disagree	872	16.5
Agree	2169	41.1
Strongly agree	1695	34.0
**Frequency of physical activity in free-time outside of school**		
Never	155	2.4
Less than once a month	123	2.1
Once a month	151	2.9
Once a week	582	11.9
2 to 3 times a week	1318	25.8
4 to 6 times a week	1595	31.6
Every day	1230	23.4

*Based on weighted N.

Table 2 provides a description of the GIS-derived neighborhood measures. Most of these measures were positively skewed. For instance, while the differences between the 5th and 50th percentile values for meadows and treed areas was <3% land coverage, the differences between the 50th and 95th percentile values were >12% land coverage and the differences between the 95th and 99th percentile values were >10% land coverage (Table 2). Because of the extreme values at the upper end of the distribution for the meadows, treed areas, developed park space, and recreational facility variables, the extreme values for these variables were truncated at the 95th percentile prior to conducting further analyses. Thus, all meadow area values greater than 15.1% and all treed area values greater than 27.2% were truncated to these values.

**Table 2:** **Selected descriptive characteristics of participants’ neighborhoods as obtained using geographic information systems**

**Variable**	**Percentile**						
	**1st**	**5th**	**25th**	**50th**	**75th**	**95th**	**99th**
Meadows (% land area)	0	0	1.0	2.4	5.6	15.1	25.7
Treed areas (% land area)	0	0	0	2.7	9.8	27.2	49.6
Park and playground space (% land area)	0	0	0.8	2.4	5.6	15.2	24.3
Recreation facilities (number)	0	0	0	1	2	6	14
Low speed roads (% road distance ≤ 50 km/h)	54.3	64.1	77.0	85.8	91.0	96.3	100
Average household income ($ CAD per year)	34,892	41,188	55,827	70,631	85,658	107,583	126,725

Correlations between the neighborhood physical environment measures are shown in Table 3. The proportion of land covered by meadows was weakly positively correlated to the proportion of land covered by treed areas (r = 0.16, p < 0.001). The meadows and treed area measures were negatively correlated with the proportion of land covered by developed parks and the number of recreational facilities (p < 0.001).

**Table 3:** **Correlations (Spearman r values) between the physical neighborhood environment measures**

	**Meadows**	**Treed areas**	**Park and playground space**	**Recreation facilities**	**Low speed roads**
**Meadows**	1.00				
**Treed areas**	.16	1.00			
**Park and playground space**	-.11	-.14	1.00		
**Recreation facilities**	-.26	-.22	.01	1.00	
**Low speed roads**	.01	.22	-.01	-.11	1.00

*Note:* all correlations stronger than 0.01 were statistically significant (p < 0.001).

The relations between the undeveloped green space measures and confounders with the frequency of being physically activity in free-time outside of school hours are shown in Table 4. The proportion of neighborhood land covered by meadows was not associated with the physical activity outcome before or after adjusting for confounders (p > 0.6). However, the proportion of neighborhood land covered by treed areas was positively associated with the physical activity outcome, and this association was significant after adjusting for confounders (p = 0.02). The final multivariate model indicated that for each additional 5% of neighborhood land covered by treed areas there was a corresponding 5% increase (95% confidence interval: 1-10% increase) in the relative odds of increasing physical activity outside of school hours. Of the confounding variables, female gender, a higher grade, non-white race, and completing the survey in the winter were associated with a lower odds of the physical activity outcome. A higher perceived neighborhood safety was associated with a higher odds of the physical activity outcome (p < 0.05).

**Table 4:** **Relations between undeveloped green spaces and frequency of being physically active in free-time outside of school hours**

	**Bivariate models,**	**Multivariate model for meadows,**	**Multivariate model for treed areas,**
	**OR (95% CI)**	**OR (95% CI)**	**OR (95% CI)**
**Undeveloped green spaces**			
Meadows (per 5% land coverage)	1.03 (0.93-1.13)	0.98 (0.87-1.08)	N/A
Treed areas (per 5% land coverage)	1.07 (1.03-1.11)	N/A	1.05 (1.01-1.10)
**Intrapersonal and family confounders**			
Gender (female vs. male)	0.64 (0.56-0.74)	0.64 (0.55-0.74)	0.64 (0.55-0.73)
Grade (per grade)	0.78 (0.73-0.85)	0.79 (0.73-0.86)	0.78 (0.72-0.85)
Race (non-white vs. white)	0.64 (0.55-0.75)	0.69 (0.56-0.83)	0.72 (0.61-0.85)
Perceived family wealth (per 1 unit change)	1.17 (1.10-1.25)	1.15 (1.09-1.22)	1.14 (1.08-1.21)
Parents in household (dual vs. single)	0.88 (0.75-1.02)	0.96 (0.81-1.24)	0.95 (0.80-1.12)
Season of survey (winter vs. fall and spring)	0.78 (0.64-0.94)	0.81 (0.68-0.98)	0.81 (0.67-0.97)
**Neighborhood confounders**			
Park and playground space (per 5% land coverage)	0.95 (0.90-1.01)	0.99 (0.94-1.05)	1.00 (0.96-1.05)
Recreation facilities (per each additional facility)	0.94 (0.90-0.99)	0.95 (0.91-0.99)	0.96 (0.92-1.02)
Perceived neighborhood safety (per 1 unit change)	1.27 (1.18-1.37)	1.22 (1.13-1.31)	1.21 (1.12-1.30)
Low speed roads (per 10% distance ≤ 50 km/h)	1.04 (0.95-1.14)	N/A	N/A
Average income (per $10,000)	1.04 (0.98-1.08)	N/A	N/A

## Discussion

The objective of this study was to determine whether the presence of undeveloped green spaces in home neighborhoods are associated with physical activity amongst grade 6 to 8 children (approximate ages 11 to 13-years-old). The key finding was that the proportion of neighborhood land area comprised of treed areas was positively associated with the frequency of physical activity performed in free-time outside of school hours. Conversely, physical activity was not associated with land area comprised of meadows, land area comprised of developed parks and playgrounds, or the number of recreation facilities.

The findings of this study build upon previous research from the Canadian HBSC which suggests that the physical activity of 11 to 13 –year-old Canadians is associated with the playability of their home neighborhood. In our experience, the playability of a neighborhood for Canadians of this age group is a function of its perceived safety [17], the presence of cul-de-sacs [7,8], and as shown in this paper, the presence of undeveloped treed areas. As shown in this paper and other more focused studies, the presence of recreational facilities [8,17] and developed park and playground space [8] in the home neighborhood are not positively associated with physical activity among older Canadian children and youth. While the presence of recreation facilities within 1 km of the home may not be important, this does not mean that the presence of these facilities is not important at longer distances or the community level, as found previously [16,18]. Children are typically driven to recreation facilities to participate in organized sports [19], which they tend to do about twice a week [20], and thus it may not be crucial for a child’s overall physical activity level to live within walking distance and actively travel to these facilities. In terms of developed parks and playgrounds, although we did not do a formal assessment, it was evident while completing our GIS measures that many of the developed parks and playgrounds were small (i.e., < ½ acre), had limited space for running around, and contained features such as slides and swings that are more relevant for younger children than the age group studied here. It has been proposed that developed parks and playgrounds are simpler, have less diversity, and are less exciting and stimulating than are natural playscapes such as treed areas [21,22].

It is noteworthy that 26% of the children in this study had no treed areas whatsoever within a 1 km distance of their home. Only 38% lived in a neighborhood were at least 5% of the land area was comprised of treed areas and only 23% lived in a neighborhood were at least 10% of the land area was comprised of treed areas. Thus, the majority of urban residing Canadian children have access to little or no treed areas within a ~10 minute walk of their home and within their independent mobility range. Independent mobility refers to the freedom children have to play and travel outdoors without adult supervision. Although this has not been assessed in Canada, data from Australia indicate that 53% of 10 to 12-year-olds have an independent mobility of less than 1 km [14]. Future research needs to examine policies and practices around the protection of existing treed areas when new housing developments are made, as well as the split between how much of the non-housing spaces is devoted to developed and undeveloped green space.

A key limitation of this study is the cross-sectional design. It is possible that parents in part select their home neighborhood because it contains features that promote physical activity for their children. This study was also limited to grade 6 to 8 children residing in urban areas. Rural areas would have included more treed and meadow areas, and if they had been included the correlations between the undeveloped green spaces and physical activity may have differed. Another limitation is that the physical activity outcome was self-reported by the child participants; this measure is likely suspect to recall error and a social desirability bias. Furthermore, the question used to assess the physical activity outcome was not ideal as it does not capture the duration and all the types and intensities of outdoor play that grade 6 to 8 children engage in. Finally, the covariate measure of neighborhood safety was based on perceived safety rather than an objective measure, although perceived safety is a stronger correlate of physical activity than objective safety measures [23].

## Conclusions

In conclusion, within this large and diverse sample of grade 6 to 8 Canadian children the proportion of land area in the home neighborhood that was comprised of treed areas was positively associated with physical activity. Thus, treed areas may contribute to the playability of a neighborhood. To our knowledge, this is the first study to document this relationship. Future research in other settings and populations and with other study designs is warranted.

## Abbreviations

HBSC: Health behaviour in school-aged children survey
GIS: Geographic information systems

## References

[1] Colley RC, Garriguet D, Janssen I, Craig CL, Clarke J, Tremblay MS (2009). Physical activity of Canadian children and youth: accelerometer results from the 2007 to, Canadian health measures survey. Health Rep.

[2] Carver A, Timperio A, Crawford D (2008). Playing it safe: the influence of neighbourhood safety on children's physical activity. A review. Health Place.

[3] Krahnstoever Davison K, Lawson CT (2006). Do attributes in the physical environment influence children's physical activity? A review of the literature. Int J Behav Nutr Phys Act.

[4] Ferreira I, van der Horst K, Wendel-Vos W, Kremers S, van Lenthe FJ, Brug J (2007). Environmental correlates of physical activity in youth - a review and update. Obes Rev.

[5] Sallis JF, Prochaska JJ, Taylor WC (2000). A review of correlates of physical activity of children and adolescents. Med Sci Sports Exerc.

[6] Owen N, Humpel N, Leslie E, Bauman A, Sallis JF (2004). Understanding environmental influences on walking; review and research agenda. Am J Prev Med.

[7] Mecredy G, Pickett W, Janssen I (2011). Street connectivity is negatively associated with physical activity in Canadian youth. Int J Environ Res Public Health.

[8] Laxer RE, Janssen I (2013). The proportion of youths’ physical inactivity attributable to neighbourhood built environment features. Int J Health Geogr.

[9] Veitch J, Salmon J, Ball K (2010). Individual, social and physical environmental correlates of children's active free-play: a cross-sectional study. Int J Behav Nutr Phys Act.

[10] Frank LD, Saelens BE, Chapman J, Sallis JF, Kerr J, Glanz K (2012). Objective assessment of obesogenic environments in youth: geographic information system methods and spatial findings from the neighborhood impact on kids study. Am J Prev Med.

[11] Lachowycz K, Jones AP (2011). Greenspace and obesity: a systematic review of the evidence. Obes Rev.

[12] Currie C, Nic Gabhainn S, Godeau E (2009). 09. The Health Behaviour in School-aged Children: WHO Collaborative Cross-National (HBSC) study: origins, concept, history and development 1982-2008. Int J Public Health.

[13] Bow CJ, Waters NM, Faris PD, Seidel JE, Galbraith PD, Knudtson ML (2004). Accuracy of city postal code coordinates as a proxy for location of residence. Int J Health Geogr.

[14] Seliske L, Pickett W, Rosu A, Janssen I (2012). Identification of the optimal geographic boundary size to use when measuring the food retail environment surrounding schools. Int J Environ Res Public Health.

[15] Veitch J, Salmon J, Ball K (2008). Children’s active free play in local neighborhoods: a behavioral mapping study. Health Educ Res.

[16] Boone-Heinonen J, Popkin BM, Song Y, Gordon-Larsen P (2010). What neighborhood area captures built environment features related to adolescent physical activity?. Health Place.

[17] Nichol ME, Janssen I, Pickett W (2009). Perceptions of neighborhood safety, not recreational facilities, are associated with adolescent physical activity. J Phys Act Health.

[18] Gordon-Larsen P, Nelson MC, Page P, Popkin BM (2006). Inequality in the built environment underlies key health disparities in physical activity and obesity. Pediatrics.

[19] Hoefer WR, McKenzie TL, Sallis JF, Marshall SJ, Conway TL (2001). Parental provision of transportation for adolescent physical activity. Am J Prev Med.

[20] Clark W (2008). Kid's sport. Canadian Social Trends.

[21] Prescott E, Weinstein CS, David TG (1987). The physical environment and cognitive development in child-care centres. Spaces for Children.

[22] Fjortoft I, Sageie J (2000). The natural environment as a playground for children: landscape description and analyses of a natural playscape. Landsc Urban Plan.

[23] Janssen I (2014). Crime and perceptions of safety in the home neighborhood are independently associated with physical activity among 11–15 year olds. Prev Med.

